# Effects of preterm birth and gestational diabetes on innate lymphoid cells (ILCs) in human breast milk

**DOI:** 10.1186/s40348-026-00255-y

**Published:** 2026-09-01

**Authors:** Malgorzata Raché, Larissa Lükewille, Antje Vogelgesang, Anja Lange, Till Ittermann, Matthias Heckmann, Johanna Ruhnau

**Affiliations:** 1 Department of Neonatology and Pediatric Intensive Care, University Medicine Greifswald, Greifswald, Germany; 2 Department of Neurology, University Medicine Greifswald, Ferdinand-Sauerbruch-Straße, Greifswald, Germany; 3 Institute for Community Medicine, Div. SHIP - Clinical Epidemiological Research, University Medicine Greifswald, Greifswald, Germany; 4 German Centre for Child and Adolescent Health (DZKJ), partner site Greifswald/Rostock, Greifswald, Germany

**Keywords:** Innate lymphoid cells (ILC), Gestational diabetes mellitus (GDM), Preterm birth, Breastmilk, Sphingosine-1-phophat-receptor (S1PR1)

## Abstract

**Background:**

Breastfeeding provides key immunological and metabolic benefits for newborns, reducing complications of preterm birth and long-term risks associated with maternal gestational diabetes mellitus (GDM). However, the mechanisms underlying these protective effects, particularly the role of maternal immune cells in breast milk, remain incompletely understood. This study aimed to investigate how preterm birth and GDM influence the composition of breast milk-derived innate lymphoid cells (ILCs) during early lactation and to explore whether these maternal cells affect neonatal immune characteristics in vitro using cord blood T cells as a model system.

**Results:**

Breast milk samples (colostrum and transitional milk) were collected within the first two weeks postpartum from mothers of term and preterm infants. Flow cytometric analysis revealed that total ILC frequencies decreased over time, with no significant differences between term and preterm deliveries. Colostrum from GDM mothers, however, contained significantly higher ILC frequencies compared with samples from normoglycemic controls, indicating that maternal metabolic status alters the immunological composition of breast milk. Cord blood T cells from GDM pregnancies showed elevated expression of the sphingosine-1-phosphate receptor 1 (S1PR1), suggesting an enhanced migratory phenotype. Co-culture experiments with breast milk-derived ILCs did not further increase S1PR1 expression or modify T cell activation markers (CD25, CD69, CTLA-4, PD-1). We emphasize that all functional assays were conducted in vitro using coculture systems with umbilical cord blood cells and were not performed directly on newborns.

**Conclusions:**

Maternal GDM modifies the composition of breast milk-derived ILCs and is associated with altered receptor expression on neonatal cord blood T cells. Nevertheless, in vitro exposure to breast milk ILCs did not enhance T cell activation, suggesting that breastfeeding may help maintain neonatal immune balance despite maternal metabolic stress. These results highlight a potential regulatory role of breast milk immune cells at the maternal–infant interface and underscore the importance of breastfeeding in mitigating pro-inflammatory programming in infants of GDM mothers.

**Supplementary Information:**

The online version contains supplementary material available at https://doi.org/10.1186/s40348-026-00255-y.

## Background

Innate lymphoid cells (ILCs) belong to the group of CD45 + cells, i.e. leukocytes. ILCs, among other immune cells generated from breast milk, are essential for defending infants and forming the immune system of the neonate [1]. In addition to growth hormones, minerals, and interleukins, breast milk also includes ILCs [2]. and other immune cell types [2–7]. ILCs can be divided into three main subtypes: ILC1, ILC2, and ILC3. ILC2 and ILC3 play protective functions by supporting tissue repair, metabolic control, and gut barrier integrity, whereas ILC1 is largely pro-inflammatory and secretes cytokines like interferon-gamma (IFN-γ). ILCs can be divided into three main subtypes: ILC1, ILC2, and ILC3. These subsets are distinguished by characteristic surface markers and cytokine profiles. ILC1 (CD127⁺ CD56⁻) primarily produce interferon-gamma (IFN-γ), ILC2 (CD294⁺) secrete cytokines such as IL-5 and IL-13, and ILC3 (CD117⁺) produce IL-17 and IL-22. Functionally, ILC2 and ILC3 play protective roles by supporting tissue repair, metabolic control, and gut barrier integrity, whereas ILC1 are largely pro-inflammatory [8–11]. These cells perform essential functions similar to T cells and express CD45+, but they are lineage-negative (Lin–), as they lack common immune cell markers such as CD3, CD11b, and CD19, and do not possess antigen-specific receptors [12].

The composition of breast milk changes throughout the postnatal period, allowing it to be classified as colostrum during the first three days postpartum, transitional milk thereafter, and mature breast milk from around two weeks postpartum. Leukocytes, which are immunologically active cells, comprise 13–70% of all cells in colostrum, but their proportion drops to less than 2% in mature breast milk [13]. As the absolute leukocyte counts fall postpartum, it is likely that the falling absolute ILC counts postpartum are subject to the same regulatory mechanism. Significant health benefits of breastfeeding include improved cognitive development, immunity to infections, and decreased risk of obesity and metabolic illnesses in offspring [14]. This is particularly important for two high-risk newborn populations: infants born to mothers with gestational diabetes mellitus (GDM), who are more likely to develop obesity, insulin resistance, and cardiometabolic diseases later in life [15, 16], and very low birth weight infants (VLBWI), who are more vulnerable to infections [17]. Breastfeeding lowers the risk of early childhood overweight in infants of GDM women [18] and is linked to a lower incidence of necrotizing enterocolitis (NEC), sepsis, and mortality in VLBWI [19, 20]. As a result, clinical recommendations for the management of infants whose mothers have GDM encourage breastfeeding [21].

Evidence from animal models indicates that elevated tissue levels of ILC1 are associated with increased insulin resistance and obesity, supporting a potential role for ILC1 in the pathogenesis of metabolic disease [22]. ILC2 and ILC3, on the other hand, are connected to protective effects [10, 11]. ILC2s have been shown to assist control weight gain, brown adipose tissue development, and glucose tolerance [23]. Depleted ILC2 mice developed obesity and glucose intolerance, which were corrected by ILC2 transfer [24]. In animal studies, ILC3 cells, as major producers of IL-22, help reduce metabolic disturbances such as insulin resistance and hyperglycemia, highlighting their role in maintaining metabolic homeostasis [11]. Although ILCs are known to play important roles in metabolic regulation, little is known about the specific functions of their subtypes in breast milk or their potential impact on newborn development.

This study aims to assess whether preterm birth or GDM alters the composition of ILCs in breast milk during lactation and how these changes might influence neonatal immune responses. Specifically, we investigate whether breast milk-derived ILC1s contribute to a pro-inflammatory state in infants of GDM mothers by modulating T cell activation and migration markers (CD25, CD69, PD-1, CTLA-4) and sphingosine-1-phosphate receptor 1 (S1PR1), which regulates T cell migration and inflammation [25–28]. Understanding the functional role of ILCs in breast milk, particularly their impact on neonatal immune systems and metabolism, is crucial for uncovering potential mechanisms that could influence long-term health outcomes in infants.

## Methods

### Study design

The study protocol was approved by the ethics committee of the University Medicine Greifswald (No. BB 026/18 and BB050/19). All patients and control individuals were aged ≥ 18 years and provided written informed consent before entering the study. The exclusion criteria were clinical mastitis and delayed onset of lactogenesis (> 72 h after birth). Additionally, mothers of newborns with perinatal infections, severe congenital malformations or chromosomal disorders were excluded.

### Classification of weight and gestational weight gain (GWG)

Maternal pre-pregnancy body mass index (ppBMI) and GWG are associated with neonatal and maternal outcome of GDM [19, 25]. Therefore, these variables are considered in the analyses. A ppBMI below 18.5 kg/m^2^ is considered underweight, one up to 25 kg/m^2^ as normal weight, a ppBMI above 25 kg/m^2^ as overweight. Obesity is defined as having a ppBMI above 30 kg/m^2^.

GWG is calculated from the final weight in pregnancy minus the pre-conceptional weight [26]. The recommendation for optimal weight gain is based on ppBMI [27] Klicken oder tippen Sie hier, um Text einzugeben (Suppl. Table 1).

### Collection of biomaterials and clinical data

We conducted prospective analyses in two cohorts to investigate the distribution of ILCs in breast milk. Cohort 1 assessed breast milk from 30 mothers from term and preterm newborn to investigate the influence of gestational age (Table 1). Cohort 2 examined breast milk from 17 mothers with and without GDM who exclusively breastfed infants born at term (Table 2). Furthermore, the influence of breast milk-derived ILCs on neonatal T cells was determined in cohort 2. The study recruited mothers admitted to the University Medicine Greifswald’s (UMG) maternity unit between August 2019 and November 2021. Clinical parameters, including maternal age, ppBMI, GDM and HbA1c levels, comorbidities, mode of delivery, and gestational age, were obtained from outpatient or inpatient records. Maternal pre-pregnancy weight and height were documented using a standardized prenatal questionnaire (prenatal booklet). Clinical data and biological specimens were collected either during the postpartum hospital stay or at scheduled follow-up visits, where breast milk and blood samples were obtained in a controlled clinical setting. For participants discharged on or before day 3 postpartum, breast milk samples were collected at home by a trained study team member. Milk samples were collected on days 3, 8, and 13 (± 1 day) postpartum, representing colostrum, transitional milk, and early mature/late transitional milk, respectively. This classification follows established definitions describing the gradual shift from colostrum (days 1–5) through transitional (days 6–14) to mature milk (from ≈ day 15).

**Table 1:** Clinical characteristics of the mothers and newborns to investigate ILC distribution in breast milk with respect to gestational age

	Term group	Preterm group	*p*-value
Mother			
*n*	20	10	-
Age [years] (M ± SD)	31.5 ± 5.5	29.4 ± 4.0	0.29
GDM	6/20	1/10	-
Pre-pregnancy BMI (M ± SD)	27.2 ± 5.1	22.6 ± 2.7	0.01
ppBMI < 25	8	8	
ppBMI ≥ 25 < 30	6	2	
ppBMI ≥ 30	6	0	
GWG outside WHO recommendations (see Suppl. Table 1)			
BMI < 25	5	6	
BMI ≥ 25 < 30	3	1	
BMI ≥ 30	2	0	
GWG ± SD, there of	11.8 ± 4.8	12.1 ± 4.8	0.89
< 10 kg	5	3	
10–16 kg	8	5	
≥ 16 kg	5	2	
Mode of birth			
Spontaneous delivery	13	3	-
Operative vaginal delivery	4	1	
Caesarean section	3	6	
Newborn			
Gestational age [wks + days] (M ± SD)	40 + 0 ± 1 + 0	32 + 6 ± 3 + 4	< 0.01
Birth weight [g] (M ± SD)	3719 ± 425	2013 ± 689	< 0.01
Sex			
male	13	7	-
female	7	3	
Apgar score (M ± SD)			
1 min	8.5 ± 1.6	8.3 ± 0.8	0.78
5 min	9.6 ± 1.0	9.4 ± 0.5	0.66
10 min	9.8 ± 0.7	9.5 ± 0.5	0.24

Preterm newborns < 37 weeks gestational age; Full-term newborns ≥ 37 weeks gestation. *M *mean, *SD *standard deviation, *GDM *gestational diabetes mellitus, *BMI *Body mass index

**Table 2:** Clinical characteristics of mothers with and without gestational diabetes (GDM) and their newborns born at term

	Control group	GDM group*	*p*-value
Mother			
*n*	10	7	
Age (years) (M ± SD)	31.5 ± 6.6	34.3 ± 6.0	0.39
Pre-pregnancy BMI (M± SD)	26.4 ± 3.4	30.0 ± 10.0	0.53
ppBMI < 25	3	2	
ppBMI ≥ 25 < 30	6	2	
ppBMI ≥ 30	1	3	
GWG outside WHO recommendations (see Suppl. Table 1)			
BMI < 25	2	0	
BMI ≥ 25 < 30	5	2	
BMI ≥ 30	1	2	
GWG ± SD, there of	12.1 ± 4.1	14.0 ± 4.4	0.10
< 10 kg	2	1	
10–16 kg	7	3	
≥ 16 kg	1	2	
Mode of birth			
Spontaneous delivery	5	4	
Operative vaginal delivery	5	3	
Caesarean section	5	3	
Newborn			
Gestational age (wks + day) (M ± SD)	39 + 2 ± 1 + 2	39 + 4 ± 1 + 2	0.93
Birth weight (g) (M ± SD)	3356 ± 615	3165 ± 584	0.54
Sex			
Male	4	4	
Female	6	4	
Apgar Score (M ± SD)			
1 min	8.4 ± 0.7	8.1 ± 0.9	0.23
5 min	9.4 ± 0.7	9.6 ± 0.5	0.35
10 min	9.6 ± 0.5	10.0 ± 0.0	0.02

*MV *mean, *SD *standard deviation, *GDM *gestational diabetes mellitus, *BMI * body mass index

*1 mother with diabetes type 1

As milk maturation may be delayed in preterm mothers [28], day 13 samples were considered to reflect the early mature stage in term and late transitional phase in preterm cases. Each mother was instructed to pump 10–40 mL of milk into a sterile container using a manual breast pump (Amaryll Manual Breast Pump, ARDO Medical, Switzerland). Samples were preferably collected during the first lactation cycle in the morning (between 8 am and 10 am) and immediately delivered to the laboratory for processing. To examine blood ILCs and investigate their association with milk ILCs, a venous blood sample (approximately 6 mL in an EDTA tube) was drawn from the mother on the day of the first milk donation. The protocol for sample processing was established prior to this study. Participants were provided with instructions for the correct use of the breast milk pump.

### Processing breastmilk

Breast milk samples (typically 10–40 mL for transitional and mature milk, and 2–10 mL for colostrum) were collected and stored at 4 °C for a maximum of 24 h before processing. Processing was conducted to ensure cell viability. The samples were diluted 1:1 with a phosphate buffered saline buffer (PBS-buffer) and centrifuged at 600xg for 20 min at 4 °C, Acceleration (Acc) 7, Deceleration (Dec) 3. The lipid layer and the aqueous supernatant were removed. The cell pellet was suspended in PBS buffer, washed twice with 10 mL of PBS buffer containing 0.5% Bovine Serum Albumin IgG-Free, Protease-Free (BSA) (Jackson ImmunoResearch), and then resuspended in 1000µL of PBS buffer. Afterwards the cells were stained as described belove.

### Processing venous blood

The venous blood of mothers, collected in BD Vacutainer^®^ EDTA tubes (BD (Becton, Dickinson and Company), New Jersey, USA) on day 3 ± 1 (at least 6 mL) was treated with an ammonium chloride-potassium lysing buffer (ACK lysis, ratio 1:10, 7 min; components: ammoniumchloride (NH4Cl) 4.010 g, potassium bicarbonate (KHCO3), 0.500 g, Ethylenediaminetetraacetic acid (EDTA) 0.010 g, 500 Aqua dest.) to induce cell swelling and rupture of membranes and obtain erythrocyte-free cell pellets for flow cytometry analysis. Next, the cells were washed three times in 20 mL PBS. The cells obtained from the breast milk and blood were aliquoted into 5 mL tubes (polystyrene round-bottom, Sarstedt AG, Germany) for immunofluorescence staining.

### Cell staining

Before staining cell suspension was washed with 2 mL PBS-buffer. Fluorescence minus one (FMO) samples were used to separate positive from negative stained cells.

For live/dead staining, Zombie NIR™ was used for ILC staining and Zombie Aqua™ for the T cell activation panel (both BioLegend, California, USA). Cells were resuspended in 100 µL PBS buffer with 1 µL Zombie dye per tube for 15 min. After blocking with 10 µL FcR-block to prevent unintended antibody staining, cells were incubated for 15 min with a cocktail of 10 anti-human monoclonal antibodies (BioLegend): anti-CD3 FITC (Clone: UCHT1), anti-CD11b FITC (Clone: M1/70), anti-CD11c FITC (Clone: 3.9), anti-CD19 FITC (Clone: HIB19) as a lineage cocktail, anti-CD45 PerCP/Cy5.5 (Clone: HI30), anti-CD56 PE/Cy7 (Clone: 5.1H11), anti-CD117 BV421 (Clone: 104D2), anti-CD127 PE (Clone: A019D5), anti-CD294 BV605 (Clone: BM16), and anti-CD336 APC (Clone: P44-8). After staining, cells were washed with 2 mL FACS buffer. The cell pellet was then resuspended in 200 µL FACS buffer with Trucount Beads (BD, New Jersey, USA) for flow cytometry analysis using a BD FACS LSRII with FACSDiva 6.2 software (BD). Data were gated using FlowJo 10 (FlowJo LLC, BD, New Jersey, USA).

### Gating strategy of ILCs

The gating strategy was the same for the blood and breast milk samples. First the doublets were eliminated using a forward and sideward scatter plot. The Trucount Beads were excluded in the SSC-A vs. FSC-A plot, so that they no longer appear in the living gate and do not falsify the cell counts there. The living cells were identified by Zombie which stains dead or severely damaged cells. After gating for CD45 positive leukocytes, innate lymphoid cells (ILCs) were defined as Lineage negative (Lin-) and CD127 + cells. Therefore, the Lin- gate was settled in a SSC-A vs. FITC (Lin-) plot. Out of the Lin- gate the CD127 + cells were determined in the SSC-A vs. CD127 plot. Next step was to classify the subpopulations of the ILCs (ILC1: CD56, ILC2: CD294+, ILC3: CD117+; CD336+/-). (Gating of ILC subpopulation Suppl. Figure 1,). Fluorescence minus one (FMO) of the CD markers CD127, CD56, CD294, CD117, and CD336 to identify the ILC subpopulations within the leukocyte population are visualized in Suppl. Figure 2. The cell types ILC1, ILC2, ILC3 and leukocyte, as well as the proportion of ILC1, ILC2 and ILC3 in CD45 + cells in breast milk and blood were examined.

### Co-culture between ILCs from breastmilk and T cells from cord blood

After delivery, 6 mL of EDTA cord blood was collected by midwives for magnetic bead T cell isolation within 2 h post-birth. Breast milk was collected at 4 days (± 1 day) postpartum for ILC isolation and co-cultured with cord blood T cells for 24 h. Peripheral blood mononuclear cells (PBMCs) were isolated using Ficoll density gradient centrifugation. After that T cells were isolated using the Pan T Cell Isolation Kit (Miltenyi Biotec) and separated with a Quattro MACS magnet (Miltenyi Biotec), achieving > 95% purity, verified by flow cytometry (Suppl. Figure 3).

T cells isolated from each mother–infant pair were cultured by seeding 100,000 cells in 200 µL X-Vivo medium supplemented with 1 µg/mL IL-2 in 96-well round-bottom plates and incubated at 37 °C with 5% CO₂. T cells were maintained in monoculture for 3–5 days corresponding to the breast milk collection period reported by the mothers and the phase of lactogenesis and IL-2 was added to the medium to ensure cell viability during this period. Co-culture experiments were initiated immediately after breast milk samples were received and processed. Breast milk ILCs were isolated using the EasySep™ Human Pan ILC Enrichment Kit (Stemcell Technologies) and further enriched using the CD127 MicroBead Kit (Miltenyi Biotec). The purity of the ILCs reached approximately 80% (Suppl. Figure 4).

Two-thirds of the counted ILCs were co-cultured with 100,000 T cells from the same mother-infant pair for 24 h at 37 °C with 5% CO2, then resuspended for cell staining. T cells cultured without ILCs were maintained under identical culture conditions and for the same incubation period as the co-culture samples and were stained simultaneously after 24 h to ensure comparability between conditions. T cell activation was analyzed using markers: anti-CD3 PerCP-Cy5.5 (Clone: OKT3), anti-CD4 FITC (Clone: SK3), anti-CD25 PE (Clone: BC 96), anti-CD69 PE-Cy7 (Clone: FN50), anti-CD152 BV421(Clone: BNI3) (BioLegend), anti-CD8 APC-Cy7 (Clone: SK1), anti-CD127 BV605 (Clone: HIL-7R-M21), and anti-CD279 BV650 (Clone: EH12) (BD Biosciences), and anti-CD363 eFluor660 (Clone: SW4GYPP) (Invitrogen). FACS measurement and data acquisition followed the established protocol, including T cell subpopulation gating (Suppl, Fig. 5).

### Statistical analyses

Statistical tests were analyzed in STATA IC 15 (STATA Corp. LLC, Texas, USA) and GraphPad prism 8.2. Normality of the data was assessed using Q–Q plots and the Shapiro–Wilk test. Variables that did not meet the assumption of normality were log-transformed to enable the use of parametric tests, as well as to improve clarity in data presentation. Equality of variances was evaluated using the Robust Test for Equality of Variances. Due to considerable variability and the presence of outliers, identifying meaningful patterns was challenging. In such cases, logarithmic transformation was applied to stabilize variance and better reveal underlying data structure. Median regression models were adjusted for maternal age; outcome variables were expressed as counts per mL. Analyses for weight gain were further adjusted for maternal pre-pregnancy BMI and gestational age. To identify the differences in cell number at different days `two sample t test´ was used between different groups.

Differences in T cell marker expression between the control and GDM groups and between pure culture and co-culture conditions were analyzed using a two-way mixed-effects model, with group (control vs. GDM) as the between-subject factor and culture condition (pure culture vs. co-culture) as the within-subject factor. When significant main effects or interactions were detected, Šídák’s multiple comparisons test was applied for post hoc analyses. In order to demonstrate a connection between the variables of maternal age, ppBMI and GWG and the absolute and relative expression of the activation markers, the Spearman test was performed. For the analysis results, p-values < 0.05 were considered significant and shown in the figures as follows: * ≙ *p* < 0.05; ** ≙ *p* < 0.01; *** ≙ *p* < 0.001.

## Results

### Study population

Tables 1 and 2 depict patients according to the 2 enrolled cohorts (for details compare method section).

### Preterm birth had no influence on postnatal regulation of ILCs

**Fig. 1:**
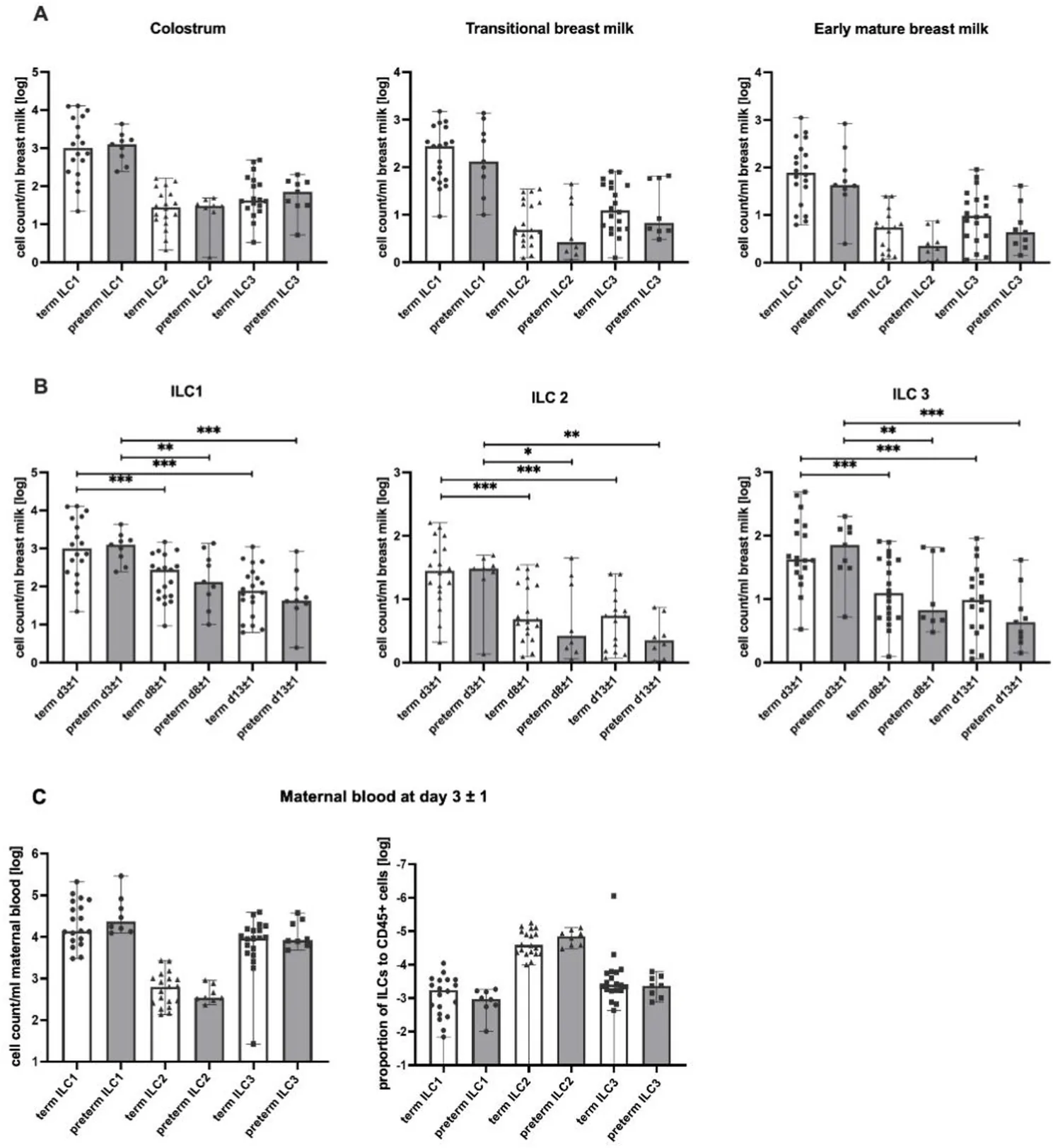
**A** Effect of gestational age: Comparison of the number of ILC1, ILC2 and ILC3 between colostrum (day 3 ± 1) and transitional breast milk (day 8 ± 1), as well as colostrum and early mature breast milk (day 13 ± 1) in term infants (n(colostrum) = 18, n(transitional breast milk) = 20, n(mature breast milk) = 20) and preterm infants (n(colostrum) = 9, n(transitional breast milk) = 9, n(mature breast milk) = 9). Statistical analysis was performed using two-sample t test. **B** Effect of postpartum age on the number of ILC 1, ILC2 and ILC3: Comparison of the number of ILC1, ILC2 and ILC3 between colostrum (day 3 ± 1) and transitional breast milk (day 8 ± 1), as well as colostrum and mature breast milk (day 13 ± 1) in term infants (n(colostrum) = 18, n(transitional breast milk) = 20, n(mature breast milk) = 20) and preterm infants (n(colostrum) = 9, n(transitional breast milk) = 9, n(mature breast milk) = 9). Statistical analysis was performed using a one-factor ANOVA with repeated measures. **B** considers the same time points as **A**. The correspondence is as mentioned in **A**. **C** Effect of gestational age in maternal blood on the number of ILC1, ILC2 and ILC3: Comparison of the absolute number and frequencies of ILC1, ILC2 and ILC3 in maternal blood (day 3 ± 1) in term infants (*n* = 19) and preterm infants (*n* = 8). Statistical analysis was performed using the two-sample t test. Time points: day 3 ± 1 (colostrum, maternal blood), day 8 ± 1 (transitional milk) and day 13 ± 1 (mature breast milk). n=number of samples. Logarithmic data with median and range are shown. *p < 0.05, **p < 0.01, ***p < 0.001

Preterm birth, adjusted for maternal age, did not alter ILCs 1–3 frequencies and absolute cell numbers at any time point in neither breast milk (Fig. 1A) nor maternal blood (Fig. 1C). To analyze changes in cell numbers over time (postnatal age), the frequencies of immune cell populations in breast milk—including ILC1, ILC2, ILC3, and total leukocytes—were determined in samples collected at day 3, day 8, and day 13 postpartum. These time points correspond to distinct stages of lactation, defined according to established criteria: colostrum (approximately days 1–5 postpartum), transitional milk (days 6–14), and early mature milk (from around day 15 onward).

The cell count of all ILC groups decreased significantly during the first two weeks of life (Fig. 1B).

### Pre-pregnancy BMI and GWG had a small effect on the number of ILC2 and ILC3

Table 3 provides median regression models to analyze associations of pre-pregnancy body mass index (ppBMI) and GWG with ILCs in breastmilk and maternal blood in term and preterm infants. Significant positive associations were found between ppBMI and ILC1 in colostrum, for ILC2 mean, and ILC3 in maternal blood, respectively. With respect to GWG, significant negative association was found for ILC3 in colostrum.

**Table 3:** Associations of pre-pregnancy body mass index (ppBMI) and weight gain with ILC frequencies

Cell population	Pre-pregnancy BMI β (95%-CI)	Gestational Weight Gain β (95%-CI)	Gestational diabetes β (95%-CI)
ILC1 colostrum	205.89 (49.68, 362.10)*	-76.74 (-311.89, 158.41)	3844.40 (613.43, 7075.37)*
ILC2 colostrum	1.97 (-0.51, 4.45)	-1.27 (-4.48, 1.94)	49.25 (11.46, 87.03)*
ILC3 colostrum	1.74 (-6.68, 10.15)	-6.71 (-12.01, -1.40)*	170.85 (41.34, 300.36)*
ILC1 blood	460.09 (-1611.74, 2531.93)	944.91 (-2100.82, 3990.64)	66552.04 (16550.50, 116553.60)*
ILC2 blood	16.82 (-47.28, 80.92)	-9.24 (-64.86, 46.39)	-214.59 (-919.25, 490.08)
ILC3 blood	617.96 (71.93, 1163.99)*	176.37 (-640.21, 992.95)	6269.26 (-7349.10, 19887.96)
Lymphocytes colostrum	46.17 (-97.45, 189.80)	-186.71 (-561.61, 188.18)	1227.71 (-1761.98, 4217.12)
Lymphocytes mean	1.64 (-62.81, 66.09)	-81.25 (-163.01, 0.51)	281.02 (-860.06, 1422.12)

A regression analysis showed that GDM influences ILC counts even when adjusted for maternal age, ppBMI, and gestational age. Results based on median regression models adjusted for maternal age. Analyses for weight gain and gestational diabetes were further adjusted for ppBMI and gestational age. *CI *confidence interval

*p < 0.05

### Gestational diabetes influenced the number/frequency of ILCs in breast milk and blood

**Fig. 2:**
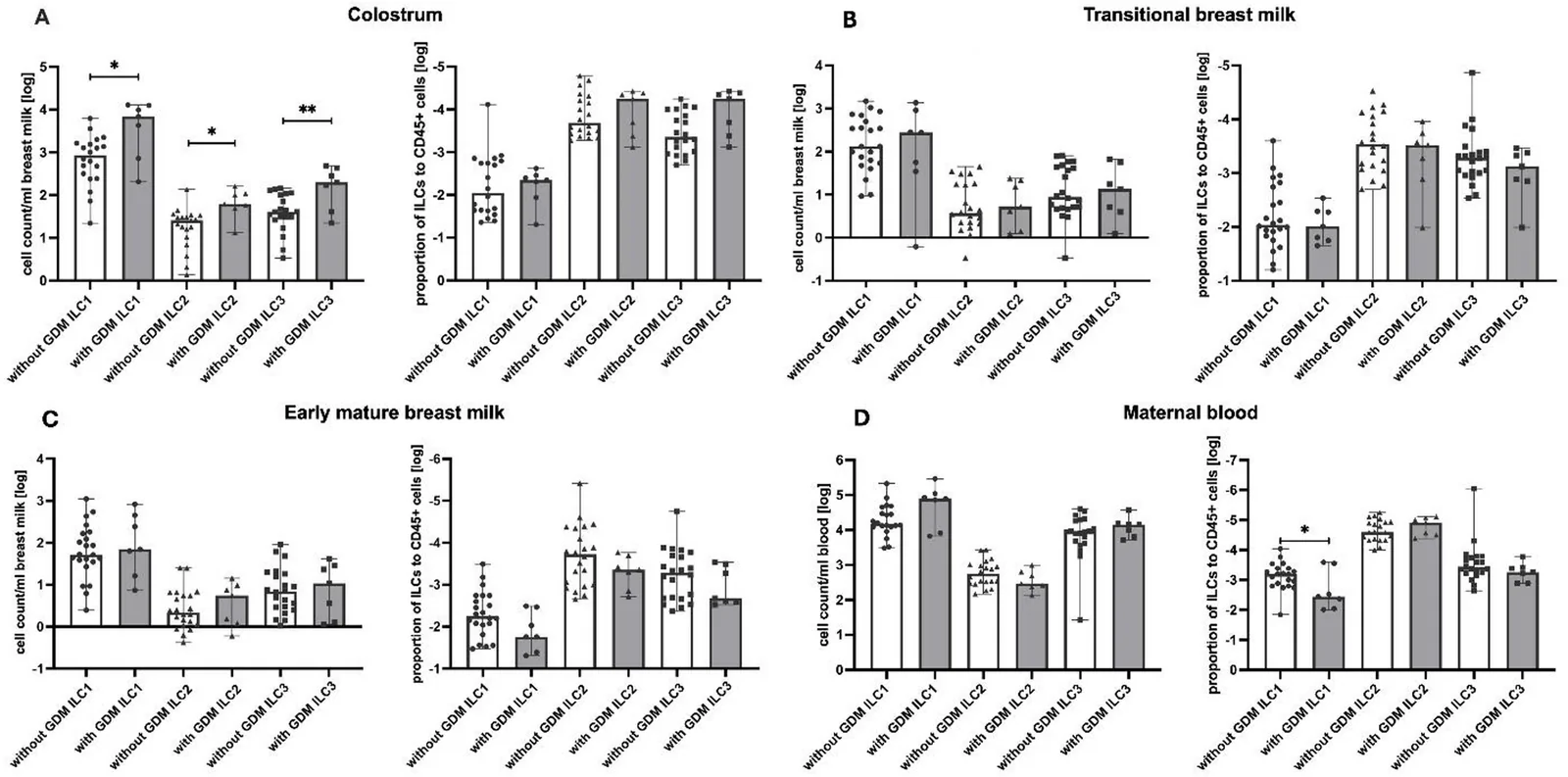
Effect of gestational diabetes (GDM) on the absolute number and frequencies of ILCs in colostrum and maternal blood. Statistical analysis was performed using the two-sample t test. **A** Comparison of the absolute number of ILCs in colostrum (day 3 ± 1) postpartum between mothers without GDM (*n* = 20) and with GDM (*n* = 7). GDM was associated with significantly increased numbers of ILC1 (*p* = 0.0125), ILC2 (*p* = 0.0109), and ILC3 (*p* = 0.0085). Comparison of the proportion of CD45⁺ ILCs in breast milk between groups without and with GDM. No significant differences were observed. **B** Comparison of the absolute number of ILCs in transitional breast milk (day 8 ± 1) postpartum between mothers without GDM (*n* = 22) and with GDM (*n* = 7). No significant differences were observed. Comparison of the proportion of CD45⁺ ILCs in breast milk between groups without and with GDM. No significant differences were observed. **C** Comparison of the absolute number of ILCs in early mature breast milk (day 13 ± 1) postpartum between mothers without GDM (*n* = 22) and with GDM (*n* = 7). No significant differences were observed. Comparison of the proportion of CD45⁺ ILCs in breast milk between groups without and with GDM. No significant differences were observed. **D** Comparison of the absolute number of ILCs in maternal blood at day 3 ± 1 postpartum between mothers without (*n* = 20) and with GDM (*n* = 7). No significant differences were observed. Comparison of the proportion of CD45⁺ ILC1 in maternal blood at day 3 ± 1, showing a higher frequency in the group without GDM (*p* = 0.0408). Logarithmic data are shown with median and range. *p < 0.05, **p < 0.01, ***p < 0.001

**Fig. 3:**
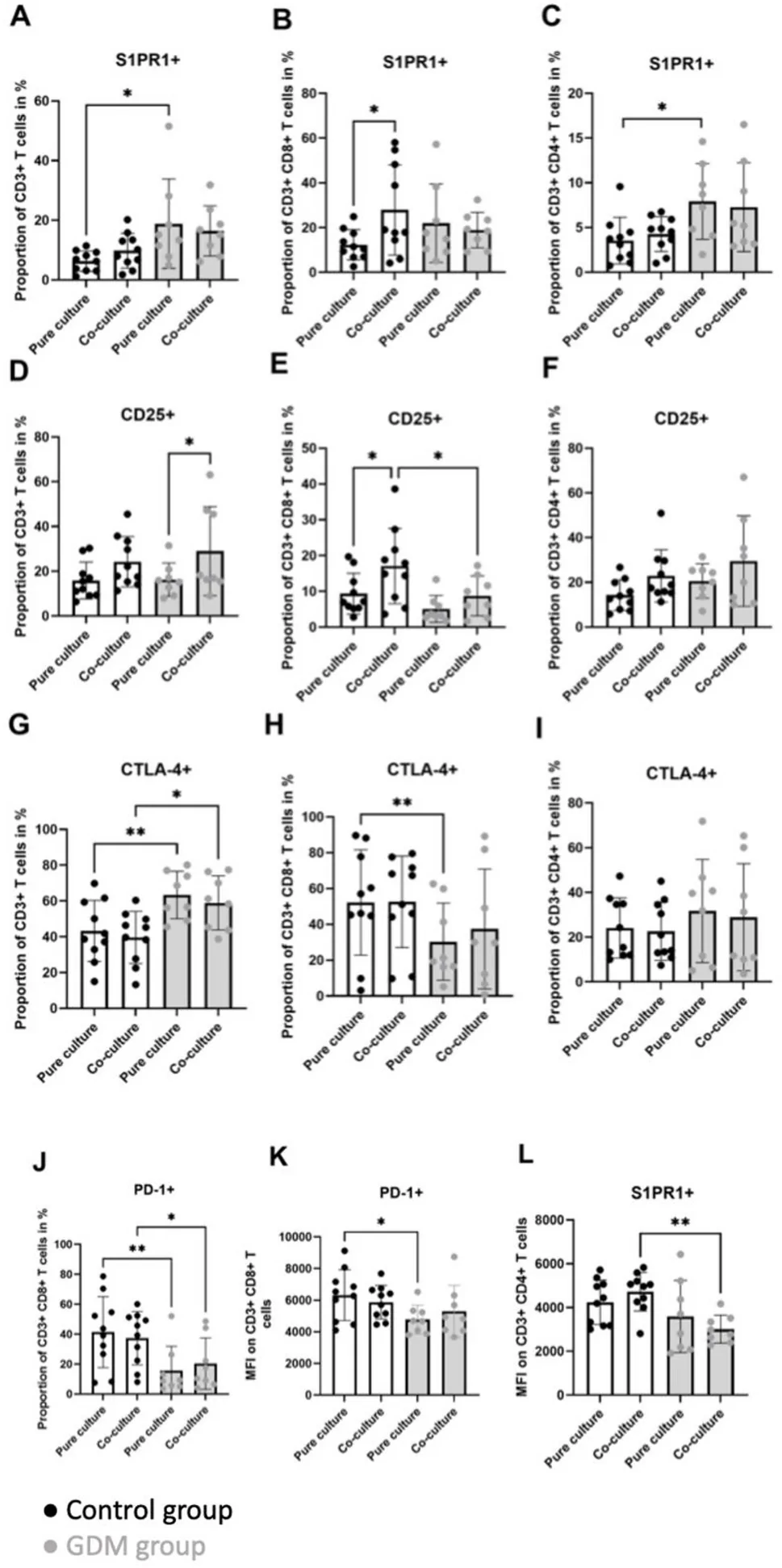
Effect of GDM and breast milk-derived ILCs on activation marker expression in neonatal T cells. Relative expression of S1PR1 (**A**–**C**), CD25 (**D**–**F**), CTLA-4 (**G**–**I**), PD-1 (**J**), and MFI of PD-1 (**K**) and S1PR1 (**L**) on CD3⁺ T cells, CD3⁺CD4⁺ T cells, and CD3⁺CD8⁺ T cells isolated from cord blood of term infants born to mothers with and without GDM. T cells were analyzed after pure culture and co-culture with breast milk-derived ILCs. Statistical analysis was performed using a two-way mixed-effects model with Šídák’s multiple comparisons test. **A** The proportion of S1PR1⁺ CD3⁺ T cells was significantly higher in the GDM group than in the control group in pure culture (*p* = 0.0104). **B** The proportion of S1PR1⁺ CD3⁺CD8⁺ T cells was significantly higher in the GDM group in pure culture (*p* = 0.0169) and showed a trend towards a decrease from pure culture to co-culture within the GDM group (*p* = 0.0615). **C** The proportion of S1PR1⁺ CD3⁺CD4⁺ T cells was significantly higher in the GDM group in pure culture (*p* = 0.0223). **D** The proportion of CD25⁺ CD3⁺ T cells was significantly increased in co-culture compared with pure culture in the GDM group (*p* = 0.047). **E** The proportion of CD25⁺ CD3⁺CD8⁺ T cells was significantly higher in co-culture than in pure culture in the control group (*p* = 0.0374) and was significantly higher in the co-culture of the GDM group than in the co-culture of the control group (*p* = 0.0234). (F) No significant differences in CD25 expression on CD3⁺CD4⁺ T cells were observed between groups or culture conditions. **G** The proportion of CTLA-4⁺ CD3⁺ T cells was significantly higher in the GDM group than in the control group in pure culture (*p* = 0.0008) and showed a similar trend in co-culture (*p* = 0.118). **H** The proportion of CTLA-4⁺ CD3⁺CD8⁺ T cells was significantly lower in the GDM group than in the control group in pure culture (*p* = 0.0063) and showed a similar trend in co-culture (*p* = 0.0864). **I** No significant differences in CTLA-4 expression on CD3⁺CD4⁺ T cells were observed between groups or culture conditions. **J** The proportion of PD-1⁺ CD3⁺CD8⁺ T cells was significantly lower in the GDM group than in the control group in both pure culture (*p* = 0.0017) and co-culture (*p* = 0.0373). **K** PD-1 MFI on CD3⁺CD8⁺ T cells was significantly lower in the pure culture of the GDM group than in the control group (*p* = 0.0276). **L** S1PR1 MFI on CD3⁺CD4⁺ T cells was significantly lower in the co-culture of the GDM group than in the control group (*p* = 0.003). Data are presented as bar graphs with median and interquartile range. Control group: *n* = 10; GDM group: *n* = 8. *p < 0.05, **p < 0.01

GDM was associated with an increase in absolute number of all three ILC populations in colostrum (ILC1 *p* = 0.0125; ILC2 *p* = 0.0109; ILC3 *p* = 0.0085) (Fig. 2A). A regression analysis showed that GDM influenced ILC counts even when adjusted for maternal age, ppBMI, and gestational age (Table 3). Neither the frequencies in breast milk nor the absolute numbers in maternal blood were altered by the influence of GDM (Fig. 2). The group without GDM showed higher frequencies of ILC1 in maternal blood (*p* = 0.0408 (Fig. 2D). No differences were seen at the later time points (Figs. 2B and C and 3).

### GDM influences S1PR1 expression and T cell activation in ILC co-culture

The proportion of S1PR1-positive CD3 + T cells was significantly higher in the pure culture of the GDM group than in the control group (*p* = 0.0104; A). A similar pattern was observed for CD3 + CD8+ T cells (*p* = 0.0169; B) and CD3 + CD4+ T cells (*p* = 0.0223; C). In the GDM group, the proportion of S1PR1-positive CD3 + CD8+ T cells showed a trend towards a decrease from pure culture to co-culture (*p* = 0.0615; B).

In the GDM group, the proportion of CD25-positive CD3 + T cells was significantly higher in co-culture than in pure culture (*p* = 0.047; D). In the control group, the proportion of CD25-positive CD3 + CD8+ T cells was significantly higher in co-culture than in pure culture (*p* = 0.0374; E). Furthermore, the proportion of CD25-positive CD3 + CD8+ T cells was significantly higher in the co-culture of the GDM group compared with the co-culture of the control group (*p* = 0.0234; E). No significant differences in CD25 expression on CD3 + CD4+ T cells were observed between groups or culture conditions (F).

The proportion of CTLA-4-expressing CD3 + T cells was significantly increased in the GDM group compared with the control group in pure cultures (*p* = 0.0008) and showed a similar trend in co-cultures (*p* = 0.118; G). In contrast, the proportion of CTLA-4-expressing CD3 + CD8+ T cells was significantly decreased in the pure cultures of the GDM group compared with the control group (*p* = 0.0063), with a similar trend observed in co-cultures (*p* = 0.0864; H). No significant differences in CTLA-4 expression on CD3 + CD4+ T cells were detected between groups or culture conditions (I).

The proportion of PD-1-expressing CD3 + CD8+ T cells was significantly reduced in the GDM group compared with the control group in both pure cultures (*p* = 0.0017) and co-cultures (*p* = 0.0373; J). The median fluorescence intensity (MFI) of PD-1 on CD3 + CD8+ T cells was significantly lower in the pure culture of the GDM group than in the pure culture of the control group (*p* = 0.0276; K). The MFI of S1PR1 on CD3 + CD4+ T cells was significantly lower in the co-culture of the GDM group than in the co-culture of the control group (*p* = 0.003; L). To provide a complete overview of the analyzed parameters, all additional datasets not shown in the main figures are included in the Supplementary Material (Supplementary Fig. 6 und 7).

**Fig. 4:**
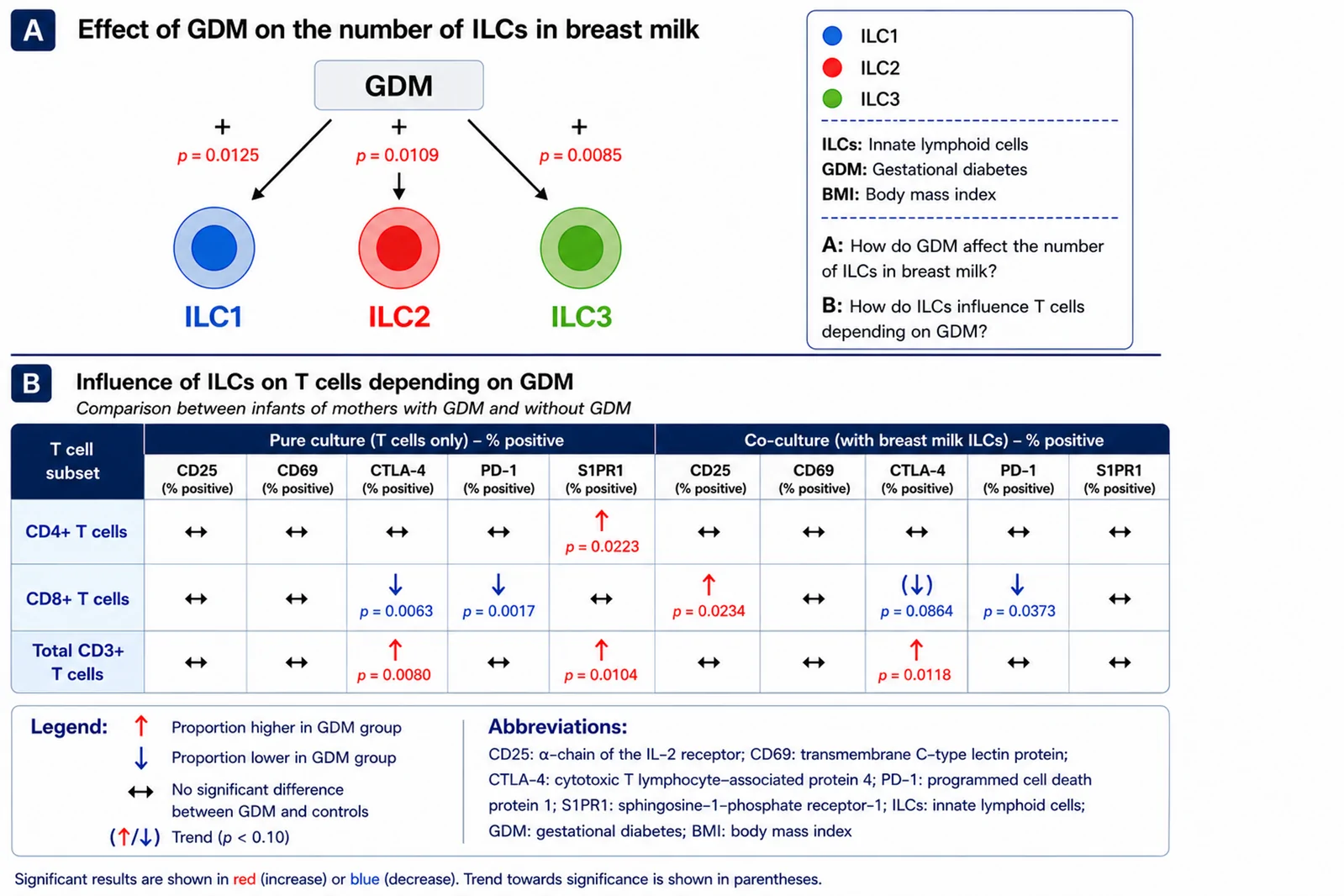
Summary illustration of the influence of gestational diabetes mellitus (GDM) on the number of innate lymphoid cells (ILCs) in human breast milk and the effects of breast milk-derived ILCs on neonatal T cells in pure and co-culture. **A** GDM was associated with significantly higher numbers of ILC1, ILC2, and ILC3 in human breast milk compared with controls. P-values for significant group differences are indicated. **B** Comparison of neonatal cord blood CD4⁺ T cells, CD8⁺ T cells, and total CD3⁺ T cells from infants of mothers with GDM and healthy controls cultured either alone (pure culture) or in co-culture with breast milk-derived ILCs. Arrows indicate a higher (↑, red) or lower (↓, blue) proportion in the GDM group compared with controls, horizontal arrows (↔) indicate no significant difference, and arrows in parentheses ((↑/↓)) indicate a trend toward significance (p < 0.10). Statistical analysis was performed using the Wilcoxon–Mann–Whitney test. Abbreviations: ILC, innate lymphoid cell; GDM, gestational diabetes mellitus; CD25, α-chain of the IL-2 receptor; CD69, transmembrane C-type lectin protein; CTLA-4, cytotoxic T-lymphocyte-associated protein 4; PD-1, programmed cell death protein 1; S1PR1, sphingosine-1-phosphate receptor-1

Figure 4 summarizes the results ILC-related outcomes stratified by GDM versus control groups.

Correlation analyses were performed to assess the relationship between maternal BMI, gestational weight gain (GWG), and maternal age and the expression of T-cell activation markers in term infants. Although several positive and negative trends were observed, no statistically significant correlations were detected between maternal characteristics and the relative or absolute expression of PD-1, CD69, S1PR1, CD25, or CTLA-4 on CD3+, CD4+, or CD8 + T-cell subsets in either the GDM or control groups (Suppl. Figure 8–10).

## Discussion

This study aims to understand how ILCs, particularly ILC1, in breast milk may affect the neonatal immune system. We investigate differences in ILC regulation between mothers of term and preterm infants, assess the impact of GDM on ILC regulation and milk composition, and explore how breast milk ILCs influence neonatal T cells, especially in infants born to mothers with GDM. The feature image summarizes the results. Our data show, for the first time, a significant decrease in the absolute number of ILCs in all subsets per mL of breast milk during the first two weeks of lactation without differences between term and preterm breastmilk. In line with the findings from Trend et al. we found no difference in leukocyte concentration between preterm and term breast milk in this period. Humoral components like IgA, lysozymes, and antimicrobial peptides may play a more crucial role in defending against acute infections [29, 30]. While preterm infants are more prone to enteric infections and intestinal inflammation in which nutrition plays an important role, this does not appear to be explained by a reduced ILC cell count in breast milk of mothers of premature babies [30].

### GDM and postnatal regulation of ILCs in breast milk and their influence on neonatal T cells

In contrast to preterm birth, GDM was associated with increased cell counts for all three ILC classes in colostrum compared to controls. While existing literature extensively discusses the effects of breastfeeding on child outcomes post-GDM, no data on ILCs are available [18, 31–33]. Although data on breast milk leukocytes in GDM are lacking, previous studies have demonstrated altered concentrations of CRP, insulin and glucose in breast milk from women with GDM, while IL-6 levels remained unchanged (Choi et al., 2022). These findings support the concept that maternal metabolic dysregulation may influence the immune composition of breast milk [34]. These observations support the concept that maternal metabolic dysregulation may also affect breast milk immune cell populations. The immune system and GDM are closely linked, with changes in maternal blood cell profiles associated with metabolic and pregnancy-related conditions. GDM is characterized by low-grade inflammation, including increased neutrophil and NK cell counts, and reduced Treg cell numbers in blood in both mice and humans [35–37]. As seen in mothers with GDM in our study, the increased levels of proinflammatory ILC1 and ILC3 in breast milk may further contribute to an inflammatory milieu in the early postpartum period.

In addition to cellular alterations, changes in breast milk metabolites may also contribute to immune modulation in the offspring. Recent studies demonstrated that long-chain saturated fatty acids in breast milk are associated with the induction of inflammatory ILC3 responses and the development of atopic dermatitis, highlighting a direct interaction between milk-derived metabolites and innate lymphoid cell function [38]. Furthermore, short-chain fatty acids (SCFAs) in breast milk have been linked to infant gut microbiota composition, immune maturation, and the subsequent risk of allergic sensitization and atopic disease [39, 40]. Since maternal metabolic disorders such as GDM are known to alter breast milk composition, including lipid and metabolite profiles, it is conceivable that metabolic changes in breast milk may directly influence ILC regulation or function and thereby contribute to long-term immune programming in the offspring.

While our co-culture experiments provide valuable insight into possible interactions between breast milk–derived ILCs and neonatal immune cells, they represent an in vitro model using cord blood T cells and cannot directly demonstrate immune effects in the offspring. Therefore, the results should be interpreted as indicative of potential cellular communication rather than functional immunomodulation in vivo. One limitation of the present study is the considerable variability in the number of breast milk-derived ILCs available for co-culture experiments, ranging from 95 to 7,650 cells per condition (mean 2,853). This variability likely reflects both biological differences between samples and technical limitations related to the low abundance of ILCs in breast milk. Unequal ILC input may have contributed to variability in T cell activation readouts and potentially reduced the sensitivity to detect subtle functional effects. Nevertheless, correlation analyses between ILC numbers and activation marker expression did not reveal significant associations, arguing against a major quantitative bias driving the observed findings. Furthermore, our study revealed a significantly higher proportion of ILC1 cells in the blood of mothers with GDM. These findings are consistent with previous observations that newborns of mothers with elevated ppBMI exhibit increased levels of proinflammatory cytokines, including IL-6, IL-8, ICAM3, and TNFR1, which show positive correlations with maternal ppBMI [41].

Castillo-Courtade et al. observed suppressed Th1 cells in Peyer’s patches and a dominant Th2 anti-inflammatory immune response in oligosaccharide-fed mice suggesting a potential link between breast milk composition and immune response [42]. We therefore investigated the interaction between breast milk derived ILCs and neonatal T cells with respect to GDM and a possible proinflammatory immune response in the offspring. ILCs are potent regulators of the local inflammatory and cytokine milieu, which directly influences T-cell trafficking, tissue retention, and activation programs [43]. Several studies have demonstrated that inflammatory signaling pathways induced during immune activation regulate the transcription factors FOXO1 and KLF2, which are central upstream regulators of S1PR1 expression in T cells. FOXO1 promotes the expression of KLF2, while KLF2 directly induces transcription of S1PR1 and other trafficking-associated molecules required for lymphocyte egress and recirculation [44]. In inflammatory environments, cytokine-mediated PI3K signaling can suppress FOXO1 and KLF2 activity, leading to reduced S1PR1 expression even in the absence of strong classical T-cell receptor activation [45, 46]. Because ILCs are major producers of cytokines such as IL-33, IL-7, IL-15, IFN-γ, and TGF-β, they may influence migration-associated signaling pathways in neighboring T cells during co-culture [46]. Our data indicate that cord blood T cells from GDM pregnancies show increased expression of S1PR1 and PD-1, which may reflect a higher migratory and activation potential. However, since these findings stem from short-term in vitro co-cultures without additional stimulation, they cannot be interpreted as evidence of a pro-inflammatory state in the neonate. In our study, GDM leads to an increase expression of S1PR1 on Th cells in cord blood, which fits in with recent literature demonstrating that inflammatory stimulation increases S1P1 activation in a mouse model [47]. In GDM cases, co-culturing with ILCs had no discernible effect on S1PR1 T cell expression; however, co-culturing with ILCs considerably boosts S1PR1 expression in controls when compared to pure culture. These findings suggest that, under the conditions tested, breast milk–derived ILCs from women with GDM did not enhance S1PR1 or activation marker expression in cord blood T cells. Thus, within the limits of this experimental setup, there is no evidence for a direct pro-inflammatory effect of maternal ILCs. This observation supports the hypothesis that breastfeeding may help buffer inflammatory influences of maternal metabolic alterations, although confirmation in in vivo models will be required [48].

Zhao et al. demonstrated that mothers with GDM have lower proportions of PD-1 + T cells (CD4+, CD8+, Tregs) in peripheral and retroplacental blood compared to healthy mothers. In the offspring, they found no significant difference in cord blood. Our study reveals that in pure culture, there are significantly fewer PD-1-expressing CD4 + T cells in cord blood from the GDM group compared to controls. Zhao et al. found no difference in CTLA-4 expression on T cells between mothers with and without GDM in various blood samples, a finding mirrored in our cord blood analysis [49]. Pendeloski et al. reported lower proportions of CTLA-4 + CD3+ CD4 + T helper cells and CD3 + CD8+ T cells in the peripheral blood of mothers with GDM in comparison to healthy controls, but this difference was not observed in our cord blood samples [50]. No disparities in CD69 expression on T cells were found in cord blood, whereas Pendeloski et al.‘s findings in peripheral blood of women with GDM shows an increase expression on CD3 + CD4+ and CD3 + CD8+ T cells [50].

### Influence of ppBMI and GWG

Pp-BMI and GWG significantly affect the composition of innate lymphoid cells (ILCs) in human breast milk, which can influence neonatal immunity and development. PpBMI and GWG are directly related, with underweight women typically experiencing greater GWG than overweight women (Suppl. Table 1) [51, 52]. So, generally speaking, the higher the ppBMI, the lower the GWG. A similar trend was observed in our study: participants with the highest ppBMI exhibited the lowest GWG, whereas those with the lowest ppBMI demonstrated the highest GWG. On average, weight gain across groups aligned with the Institute of Medicine (IOM) recommendations [27]. The inverse relationship between ppBMI and GWG may explain the observed increase in ILCs with higher ppBMI and lower GWG. Notably, excessive GWG is linked to adverse maternal and neonatal outcomes [19, 25]. ILCs, including ILC1, ILC2, and ILC3, play diverse roles in this context. ILC1s are associated with obesity and insulin resistance risks, while ILC2 and ILC3 may offer protective effects against these conditions [10, 22]. In our study, the breast milk of mothers with higher ppBMI or less GWG contains higher concentrations of ILC2 and ILC3 which might suggest a potential anti-inflammatory benefit for the infant. This relationship is thought to counteract the proinflammatory immune status associated with obesity. Research also indicates that the composition of cytokines in breast milk, such as IL-2, IL-6, and IL-10, varies with maternal obesity, affecting neonatal immune development [53, 54]. With respect to our findings in GDM, the proportion of mothers with ppBMI > 25 and excessive GWG were not different between the GDM group and controls. However, the significance of these trends and their impact on infant BMI development remains to be fully elucidated due to varying results across studies and the need for larger subject pools for statistical validation.

## Conclusion

This study investigated how innate lymphoid cells (ILCs) in breast milk may influence neonatal immune regulation, focusing on differences between mothers of term and preterm infants and the impact of GDM on ILC composition and neonatal T-cell characteristics. GDM was associated with higher counts of all ILC subsets in colostrum and increased proportions of ILC1 in maternal blood, indicating that maternal metabolic conditions affect immune cell profiles in breast milk. Cord blood T cells from GDM pregnancies showed elevated S1PR1 expression, reflecting enhanced migratory potential; however, this in vitro observation should not be interpreted as an indicator of neonatal inflammation. Co-culture with breast milk ILCs did not increase S1PR1 expression in the GDM group, suggesting no pro-inflammatory activation under these experimental conditions. While these findings imply a potential modulatory role of breast milk ILCs at the maternal–infant interface, causal effects on infant immunity cannot be inferred. Moreover, ILC numbers in breast milk decreased during the first two weeks postpartum, with no differences between term and preterm samples. Further in vivo studies are required to clarify the functional implications of maternal ILCs for neonatal immune development.

## Supplementary Information


Supplementary Material 1.


## Data Availability

The datasets used and/or analyzed during the current study are available from the corresponding author on reasonable request.
