# Supplementary material for: Effects of preterm birth and gestational diabetes on innate lymphoid cells (ILCs) in human breast milk
Source: Mol Cell Pediatr. 2026 Sep 1;13:45. doi: 10.1186/s40348-026-00255-y (PMC13534394; doi:10.1186/s40348-026-00255-y)
Supplement: Supplementary file 1 [file 40348_2026_255_MOESM1_ESM.docx]

**Supplementary Table 1**: Overview of weight gain during pregnancy according to the 2009 Institute of Medicine (IOM) recommendations [32].

| Pre-pregnancy BMI | BMI (kg/m^2^) | GWG (kg) |
| --- | --- | --- |
| underweight | under 18.5 | 12.5-18 |
| normal weight | 18.5 to 24.9 | 11.5 to 15.9 |
| overweight | 25.0 to 29.9 | 7 to 11.5 |
| adipositas (all grades) | over 30 | 5-9 |

**Supplemental Figures:**

**Supplementary_Figure_1:** Gating strategy for analysis of ILCs from breast milk and maternal blood. Panels **A-L** show the individual successive steps in the gating of the ILCs. SSC=Sideward Scatter, FSC=Forward Scatter, Lin-= negative for the Lineage markers CD3, CD11b, CD11c and CD19.

**Supplementary_Figure_2:** Fluorescence minus one (FMO) of the CD markers CD127, CD56, CD294, CD117, and CD336 to identify the ILC subpopulations within the leukocyte population.

**Supplementary_Figure_3:** Purity control of T cells after magnetic isolation

**Supplementary_Figure_4:** Purity control of ILC isolated from breast milk

**Supplementary_Figure_5**: Gating strategy for analysis of T cell activation after pure and coculture. SSC=Sideward Scatter, FSC=Forward Scatter.

**Supplementary_Figure_6:** Effect of GDM on activation markers on neonatal-T-cells. Relative expression of activation markers CD25, CD69, CTLA-4, PD-1 and S1PR1 on CD3⁺ T cells, CD3⁺CD4⁺ T cells and CD3⁺CD8⁺ T cells of mature infants born to mothers with and without GDM. Expression was measured after pure culture and co-culture with breastmilk-derived ILCs. Statistical analysis was performed using a two-way mixed-effects model with Šídák’s multiple comparisons test. Data are presented as bar graphs with median and interquartile range. Control group: n = 10; GDM group: n = 8. *p < 0.05, **p < 0.01.

**:**

**Supplementary_Figure_7:** Effect of GDM on activation markers on neonatal-T-cells. Absolut expression of activation markers CD25, CD69, CTLA-4, PD-1 and S1PR1 on CD3⁺ T cells, CD3⁺CD4⁺ T cells and CD3⁺CD8⁺ T cells of mature infants born to mothers with and without GDM. Expression was measured after pure culture and co-culture with breastmilk-derived ILCs. Statistical analysis was performed using a two-way mixed-effects model with Šídák’s multiple comparisons test. Data are presented as bar graphs with median and interquartile range. Control group: n = 10; GDM group: n = 8. *p < 0.05, **p < 0.01.

**Supplementary_Figure_8:** Correlation of the relative and absolute expression of the activation markers S1PR1 (**A+B**), CD69 (**C**), PD-1 (**D**) determined by flow cytometry on CD3+T cells, CD3+ CD4+ T helper cells and CD3+ CD8+ T cells of term infants after pure culture and after coculture with ILCs and the BMI of the mother. Statistical analysis was performed using the Spearman test. Control group *n*=10, GDM group *n*=8. *≙*p*<0.05, **≙*p*<0.01.

**Supplementary_Figure_9:** Correlation of the relative and absolute expression of the activation markers CD25 (**A+C+D**) and CTLA-4 (**B**) determined by flow cytometry on CD3+T cells, CD3+ CD4+ T helper cells and CD3+ CD8+ T cells of term infants after pure culture and after coculture with ILCs and the GWG of the mother. Statistical analysis was performed using the Spearman test. Control group *n*=10, GDM group *n*=8. *≙*p*<0.05, **≙*p*<0.01.

**Supplemantary_Figure_10:** Correlation of the relative and absolute expression of the activation markers PD-1 (**A**), CD69 (**C+D**) and S1PR1(**B+E**) determined by flow cytometry on CD3+T cells, CD3+ CD4+ T helper cells and CD3+ CD8+ T cells of term infants after pure culture and after coculture with ILCs and the maternal age. Statistical analysis was performed using the Spearman test. Control group *n*=10, GDM group *n*=8. *≙*p*<0.05, **≙*p*<0.01.
